# Catalpol Modulates Lifespan via DAF-16/FOXO and SKN-1/Nrf2 Activation in *Caenorhabditis elegans*

**DOI:** 10.1155/2015/524878

**Published:** 2015-03-02

**Authors:** Hyun Won Seo, Se Myung Cheon, Myon-Hee Lee, Hong Jun Kim, Hoon Jeon, Dong Seok Cha

**Affiliations:** ^1^Department of Oriental Pharmacy, College of Pharmacy, Woosuk University, Jeonbuk 565-701, Republic of Korea; ^2^Department of Medicine, Hematology/Oncology Division, Brody School of Medicine, East Carolina University, Greenville, NC 27834, USA; ^3^Department of Korean Medical Prescription, College of Korean Medicine, Woosuk University, Jeonbuk 565-701, Republic of Korea

## Abstract

Catalpol is an effective component of rehmannia root and known to possess various pharmacological properties. The present study was aimed at investigating the potential effects of catalpol on the lifespan and stress tolerance using *C. elegans* model system. Herein, catalpol showed potent lifespan extension of wild-type nematode under normal culture condition. In addition, survival rate of catalpol-fed nematodes was significantly elevated compared to untreated control under heat and oxidative stress but not under hyperosmolality conditions. We also found that elevated antioxidant enzyme activities and expressions of stress resistance proteins were attributed to catalpol-mediated increased stress tolerance of nematode. We further investigated whether catalpol's longevity effect is related to aging-related factors including reproduction, food intake, and growth. Interestingly, catalpol exposure could attenuate pharyngeal pumping rate, indicating that catalpol may induce dietary restriction of nematode. Moreover, locomotory ability of aged nematode was significantly improved by catalpol treatment, while lipofuscin levels were attenuated, suggesting that catalpol may affect age-associated changes of nematode. Our mechanistic studies revealed that *mek-1, daf-2, age-1, daf-16*, and *skn-1* are involved in catalpol-mediated longevity. These results indicate that catalpol extends lifespan and increases stress tolerance of *C. elegans* via DAF-16/FOXO and SKN-1/Nrf activation dependent on insulin/IGF signaling and JNK signaling.

## 1. Introduction

Aging is a universal biological process in all living organisms that is influenced by environmental, nutritional, and genetic factors [1]. Although mankind has already concerned with delaying aging and keeping ourselves young from the ancient time, it had been believed that the development of antiaging drug is not feasible. However, increasing lines of evidence demonstrated the pharmacological intervention in the aging process. In recent years, antiaging studies on the traditional herbal medicine have received increasing attention, because they are promising candidates for the treatment of various aging-associated diseases. Indeed, many natural products and their active compounds such as ginsenoside, curcumin, and resveratrol are known to protect our body and give a positive effect against aging in mammals as well as invertebrates through different mechanisms [2–4].

**Figure 1:**
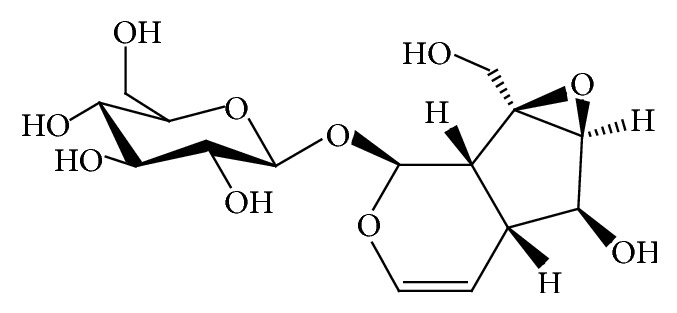
The structure of catalpol.

*Rehmannia glutinosa* has been widely used for the treatment of aging-related diseases as a traditional herbal medicine in Korea and China. Catalpol is an important iridoid glucoside with the molecular formula of C_15_H_22_O_10_ which is prevalent in the roots of* Rehmannia glutinosa* (Figure 1). Previous research showed that catalpol is effective in various age-related degenerative diseases including cancer, stroke, cognition deficit, diabetes, Alzheimer's disease, and Parkinson's disease [5–9]. These studies suggest that catalpol has therapeutic potential against aging. However, the scientific evidence on the antiaging effect of catalpol awaits identification.

Accordingly, the present study was designed to investigate the longevity effects of catalpol using* Caenorhabditis elegans* model system.* C. elegans* has become a widely accepted model for work on aging research due to their short lifespan, morphological, simplicity, ease of maintenance, and genetic manipulation [10]. In addition, many previous papers noted that compounds which have lifespan extension properties in* C. elegans* can be applicable for the treatment of cancer and neurodegenerative diseases in humans, indicating this nematode model provides an excellent environment for identifying drugs for prolonging human lifespan [11, 12]. Herein, we dissected the effects of catalpol on the lifespan and survival rate under normal and stress conditions. Furthermore, to verify the underlying pharmacological mechanisms, catalpol-mediated changes in antioxidant enzyme activities, aging related factors, and lifespan of knockout mutant strains were employed.

## 2. Materials and Methods

### 2.1. Chemicals,* C. elegans* Strains, and Maintenance

Catalpol was purchased from Sigma-Aldrich (St. Louis, MO, USA). To prepare plates supplemented with catalpol, the stock solution in dimethyl sulfoxide (DMSO) was inserted into autoclaved NGM plates (at 50°C). A final DMSO concentration of 0.1% (v/v) was maintained under all conditions. Bristol N2 (wild-type) and* Escherichia coli* OP50 strain were kindly provided by Dr. Myon-Hee Lee (East Carolina University, NC, USA). All other strains were obtained from the Caenorhabditis Genetic Center (CGC; University of Minnesota, Minneapis, MN). The transgenic strain CF1553 (*muIs84*) was used to visualize SOD-3 expression. Mechanistic study was performed using several null mutant strains including GR1307 (*mgDf50*), VC199 (*ok434*), EU1 (*zu67*), DR1572 (*e1368*), TJ1052 (*hx546*), and FK171 (*ks54*). The worms were grown at 22°C on nematode growth medium (NGM) agar plate with* E. coli* OP50 as described previously [13].

### 2.2. Lifespan Assay

The lifespan assays were performed using mutants as well as wild-type at least 3 times independently at 22°C. To obtain age-synchronized nematodes, eggs were transferred to NGM plate in the absence or presence of 6.25, 12.5, and 25 *μ*M of catalpol after embryo isolation or on the 7th day of adulthood, respectively. Test worms were considered dead when they failed to respond to prodding with the tip of a platinum wire [14]. The worms were transferred to fresh NGM plate every 2 days.

### 2.3. Assessment of Stress Resistance

The age-synchronized N2 worms were bred on NGM agar plates with or without various concentrations of catalpol. For the heat tolerance assay the adult day 4 worms were transferred to fresh plates and then incubated at 36°C. The survival rate was scored as previously described [15]. Oxidative stress tolerance was assessed as described previously with minor modification [16]. Briefly, the adult day 4 worms were subjected to plate containing 60 mM paraquat and then survivals were recorded over 30 h. Resistance to osmotic stress was measured by placing the adult day 4 worms to NGM agar plate containing 500 mM NaCl [17]. Survival rate of the worms was calculated after 12 h incubation. The survival of worms was determined touch-provoked movement. Worms which failed to respond to gentle touch with a platinum wire were considered to be dead. Each test was performed at least 3 times.

### 2.4. Measurement of SOD Activities

To assess enzymatic activity, the worm homogenates were prepared. Briefly, the wild-type worms were harvested from plate with M9 buffer on the adult day 5 and washed 3 times. Then, the collected worms were resuspended in homogenization buffer (10 mM Tris-HCl, 150 mM NaCl, 0.1 mM EDTA, and pH 7.5) and homogenized on ice. SOD activity was measured spectrophotometrically analysing the decolorization of formazan using enzymatic reaction between xanthine and xanthine oxidase. The reaction mixture contained 20 *μ*L of worm homogenates, 480 *μ*L of 1.6 mM xanthine, and 0.48 mM nitroblue tetrazolium (NBT) in 10 mM phosphate buffer (pH 8.0). After preincubation at room temperature for 5 minutes, the reaction was initiated by adding 1 mL of xanthine oxidase (0.05 U/mL) and incubation at 37°C for 20 min. The reaction was stopped by adding 500 *μ*L of 69 mM SDS, and the absorbance at 570 nm was measured. The SOD activity was expressed as a percentage of the scavenged amount per control.

### 2.5. Analysis of Intracellular ROS

Intracellular reactive oxygen species (ROS) in the nematodes was measured using molecular probe 2′,7′-dichlorodihydrofluorescein diacetate (H_2_DCF-DA). Equal number of wild-type worms was incubated in the absence or presence of catalpol. On the 4th day of adulthood, animals were exposed to NGM agar plate containing 30 mM paraquat for 3 h. Subsequently, 5 worms were transferred into the wells of a 96-well plate containing 50 *μ*L of M9 buffer. Immediately after addition of 50 *μ*L of 25 *μ*M H_2_DCF-DA solution resulting in a final concentration 12.5 *μ*M, basal fluorescence was quantified in a microplate fluorescence reader at excitation 485 nm and emission 535 nm. Plates were read every 30 min for 2 h.

### 2.6. Measurement of Aging-Related Factors

The age-synchronized N2 worms were bred on NGM agar plates with or without various concentrations of catalpol. On the 4th days of adulthood, single worms were transferred to fresh plate and their pharynx contractions were counted under an inverted microscope for 1 min. For the reproduction assay, N2 worms were raised from embryo as in the lifespan assay. L4 larvae were individually transferred to the fresh plate every day to distinguish the parent from the progeny. The progeny was counted at the L2 or L3 stage. For the growth alteration assay, photographs were taken of adult day 4 worms, and the body length of each animal was analyzed by the Nikon software (Nikon, Japan). All the tests were repeated at least 3 times.

### 2.7. Measurement of Body Movement

The age-synchronized N2 worms were bred on NGM agar plates with or without various concentrations of catalpol. On the 7th days of adulthood, single worms were transferred to fresh plate and their body movements were recorded under an inverted microscope for 20 seconds. The body movements of animals were analyzed by Nikon image software and data was expressed as total travel distance and average speed.

### 2.8. Fluorescence Microscopy and Visualization

The age-synchronized transgenic nematodes including CF1553 containing a SOD-3::GFP reporter were maintained in the presence or absence of catalpol. On the 3rd days of adulthood, nematodes were exposed to heat shock at 36°C for 2 h and allowed to recover at 22°C for 4 h. Prior to microscopy observation, transgenic animals were anesthetized with sodium azide (2%) and mounted on 2% agarose pad. The GFP fluorescence of GFP-expressing populations was directly observed under a fluorescence microscope (Nikon Eclipse Ni-u, Japan). To determine the protein expression levels, photographs of the transgenic worms were taken and assayed using ImageJ software. All experiments were done in triplicate.

### 2.9. Data Analysis

The data from the lifespan assay and stress resistance assays were plotted using Kaplan-Meier analysis and statistical significance was analyzed by log-rank test. Other data were presented as mean ± standard deviation or standard error of the mean, as indicated. Statistical significance of differences between the control and treated groups was analyzed by one-way analysis of variance (ANOVA).

## 3. Results

### 3.1. Effect of Catalpol on Lifespan-Extension and Stress Resistance

**Figure 2:**
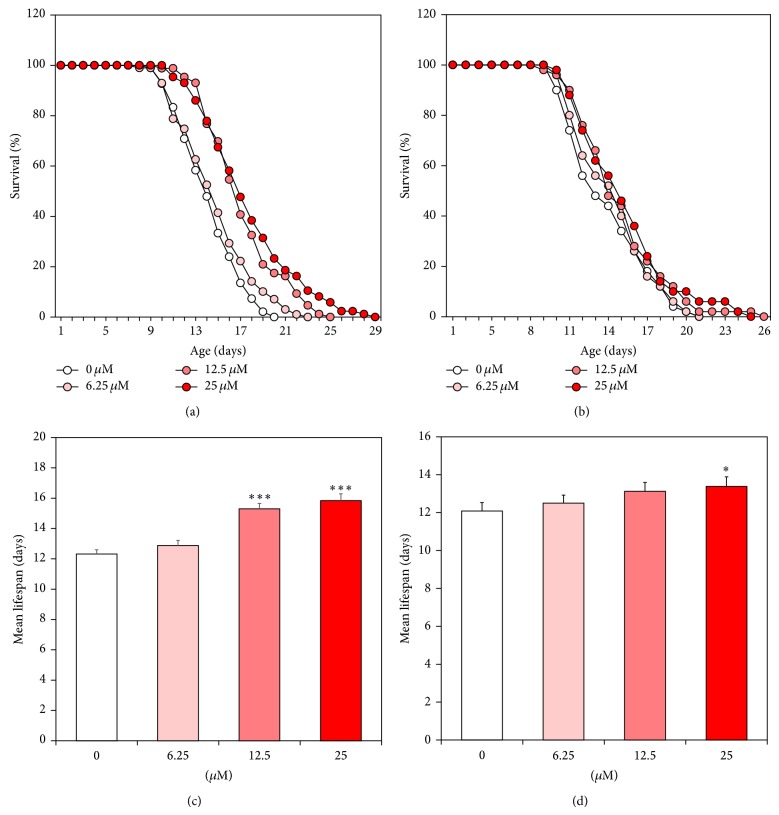
Effects of catalpol on the lifespan of wild-type N2 nematodes. Worms were grown in the NGM agar plate at 22°C in the absence or presence of catalpol after embryo isolation or 7th day of adulthood, respectively. The number of worms used per each lifespan assay experiment was 36–57 and three independent experiments were repeated (*N* = 3). The mortality of each group was determined by daily counting of surviving and dead animals. The lifespan of catalpol-treated worms after embryo isolation (a) and 7th day of adulthood (b) was plotted as a survival curve. The mean lifespan of the catalpol-treated worms after embryo isolation (c) and 7th day of adulthood (d) was calculated from the survival curves in (a) and (b), respectively. Statistical difference between the curves was analyzed by log-rank test. Error bars represent the standard error of mean (S.E.M.). Differences compared to the control were considered significant at ^*^ *P* < 0.05 and ^***^ *P* < 0.001 by one-way ANOVA.

**Figure 3:**
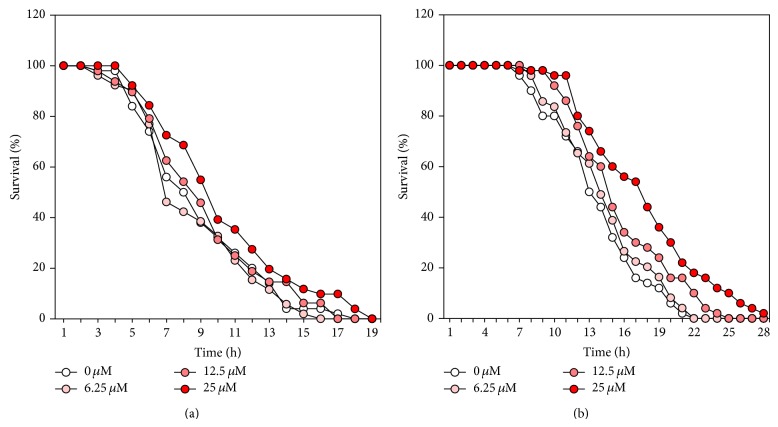
Effects of catalpol on the stress tolerance of wild-type N2 nematodes. (a) To assess thermal tolerance, worms were incubated at 36°C and then their viability was scored. (b) For the oxidative stress assays, worms were transferred to NGM agar plate containing 60 mM of paraquat, and then their viability was scored. Statistical difference between the curves was analyzed by log-rank test.

To determine the lifespan-extension properties of catalpol, lifespan assays were performed using wild-type worms. Herein, we found a concentration-dependent effect of catalpol on longevity (Figures 2(a) and 2(c)). In addition, there was a significant increase (28.5% at 25 *μ*M of catalpol, *P* < 0.001) in the estimated mean life of the catalpol-treated worms compared to control worms (Table 1). To address the possibility that catalpol may shift worm's lifespan independent of affecting developmental stage of worms, lifespan assay was conducted again using aged infertile worms (7 days of adulthood) in the presence or absence of catalpol. Interestingly, catalpol-fed aged worms displayed dose-dependent increase in lifespan, though being not as drastically as compared to catalpol exposure after embryo isolation (Figures 2(b) and 2(d)). Then we determined whether catalpol has protective effects on three different kinds of stress conditions including thermal, oxidative, and osmotic stress using wild-type worms. As can be seen in Figure 3(a), thermotolerance was elevated as a result of catalpol treatment and consequently increased survival rate dose-dependently. Furthermore, the results showed that catalpol-treated worms lived longer than control worms under 60 mM paraquat-induced oxidative stress (Figure 3(b)). However, in the case of hypertonic stress assay, catalpol failed to increase the resistance to osmotic stress (Data not shown).

### 3.2. Effect of Catalpol on Antioxidant Enzymes and Intracellular ROS Levels

**Figure 4:**
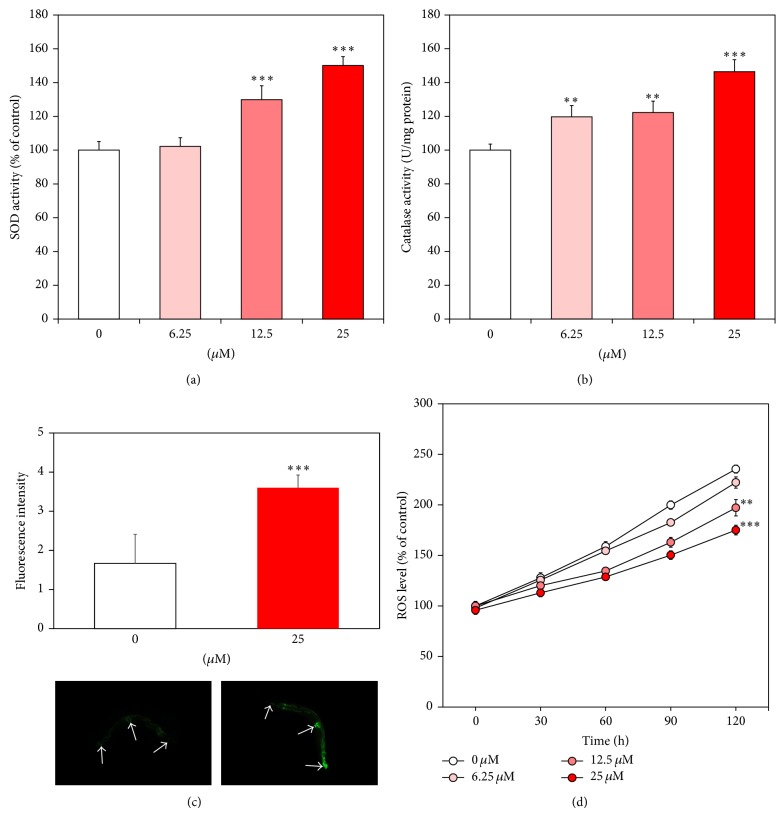
Effects of catalpol on the antioxidant enzyme activity and intracellular ROS levels of wild-type N2 nematodes. (a) The enzymatic reaction of xanthine with xanthine oxidase was used to generate •O_2_^−^ and the SOD activity was estimated spectrophotometrically through formazan formation by NBT reduction. SOD activity was expressed as a percentage of the scavenged amount per control. (b) Catalase activity was calculated from the concentration of residual H_2_O_2_, as determined by a spectrophotometric method. Catalase activity was expressed in U/mg protein. (c) Fluorescence intensity of SOD-3::GFP expression in control and catalpol-treated CF1553 worms and their images. Mean GFP intensity was represented as mean ± S.E.M. of values from 19–24 animals per each experiment (*N* = 3). (d) Intracellular ROS accumulation was quantified spectrometrically at excitation 485 nm and emission 535 nm. Plates were read every 30 min for 2 h. Data are expressed as the mean ± S.E.M. of three independent experiments (*N* = 3). Differences compared to the control were considered significant at ^**^ *P* < 0.01 and ^***^ *P* < 0.001 by one-way ANOVA.

To verify the possible mechanism of catalpol-mediated lifespan extension and elevated stress tolerance, activities and expressions of stress resistance proteins were investigated. In the present study, we measured activities of antioxidant enzymes such as superoxide dismutase (SOD) and catalase using prepared worm homogenates. As noted in Figures 4(a) and 4(b), both of them were significantly upregulated in the presence of catalpol. 25 *μ*M of catalpol increased SOD and catalase activities about 50.1% and 46.4%, respectively (*P* < 0.001). We also quantified* sod-3* gene expressions using transgenic strain CF1553 (*sod-3p::gfp* +* rol-6*). As can be seen in Figure 4(c), catalpol treatment strongly enhanced the GFP intensity of nematode in head, tail, and around vulva compared to vehicle-treated control. Our results showed that catalpol increases* sod-3* gene expression, suggesting that catalpol is an activator of SOD. Then we evaluated the influence of catalpol on the accumulation of intracellular ROS using H_2_DCF-DA probe.  Figure 4(d) shows diminished intracellular ROS level in the catalpol-fed nematode by 44.57% at 25 *μ*M (*P* < 0.001) compared to control.

### 3.3. Effect of Catalpol on Aging-Related Factors

**Figure 5:**
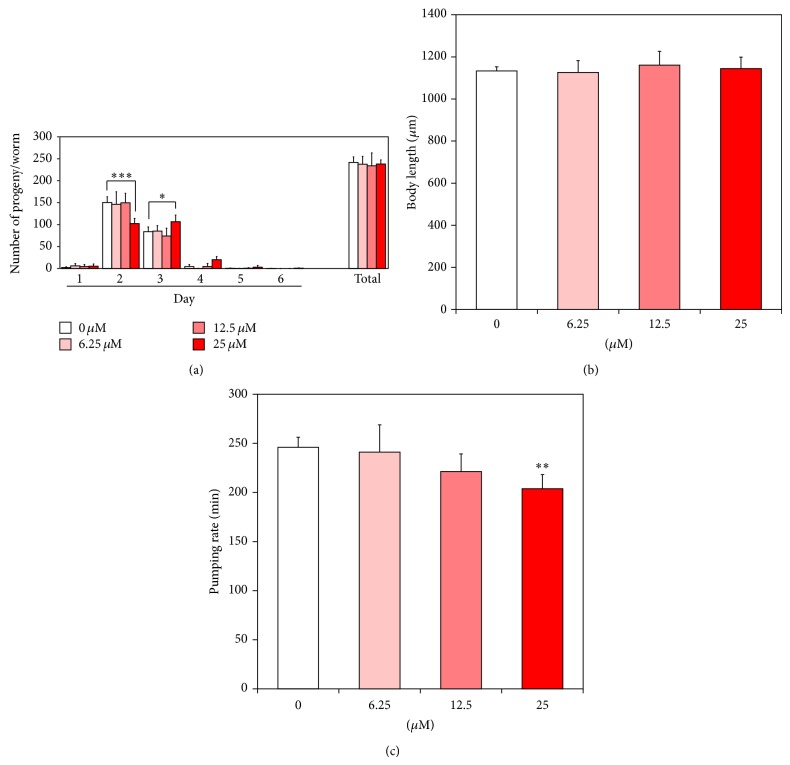
Effects of catalpol on the various aging-related factors of wild-type N2 nematodes. (a) Daily and total reproductive outputs were counted. The progeny was counted at the L2 or L3 stage. (b) For the growth alteration assay, photographs were taken of worms and the body length of each animal was analyzed. (c) On the 4th day of adulthood, the pharyngeal pumping rates. Data are expressed as the mean ± S.E.M. of three independent experiments (*N* = 3). Differences compared to the control were considered significant at ^*^ *P* < 0.05, ^**^ *P* < 0.01, and ^***^ *P* < 0.001 by one-way ANOVA.

Previous studies have suggested that longevity is closely interconnected with reproduction, food intake, and growth in many species including* C. elegans* [18–20]. Here in this work, we showed that catalpol treatment failed to alter the total progeny number, while egg laying of worms was delayed at the maximum concentration, indicating that catalpol might affect germline signaling results in delayed spermatogenesis or oogenesis (Figure 5(a)). In addition, no significant change in body length of worms was detected after catalpol exposure, suggesting that catalpol's activity is independent of growth as well as fertility (Figure 5(b)). Then, we measured the number of pharyngeal pumping to estimate the food intake of worms. As shown in Figure 5(c), the number of pharyngeal contractions declined gradually with increasing age and this age-associated diminishment was further attenuated by catalpol treatment. Based on this result, we could estimate the probability of dietary restriction like effects by catalpol.

### 3.4. Effect of Catalpol on Body Movements and Lipofuscin Accumulation

**Figure 6:**
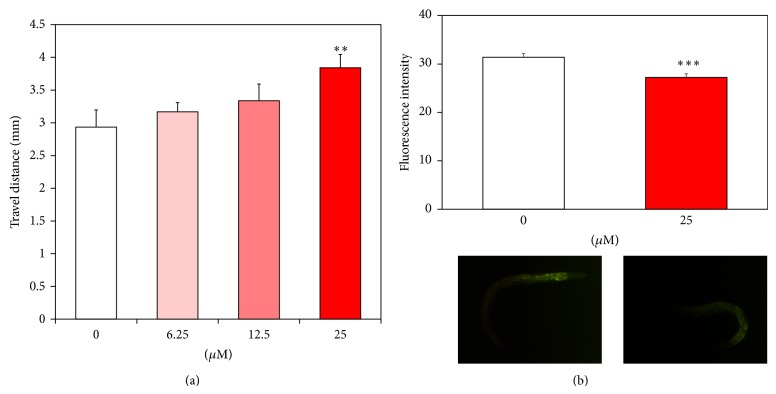
Effects of catalpol on the body movement and lipofuscin accumulation in wild-type N2 nematodes. (a) Body movement of worms were counted under a dissecting microscope for 1 min. (b) Fluorescence intensity of lipofuscin and autofluorescence image worms on the 8th day of adulthood. The fluorescence intensity was quantified using ImageJ software by determining average pixel intensity. Mean fluorescence intensity of lipofuscin was represented as mean ± S.E.M. of values from 18-19 animals per each experiment (*N* = 3). Differences compared to the control were considered significant at ^**^ *P* < 0.01 and ^***^ *P* < 0.001 by one-way ANOVA.

Then, we evaluated whether catalpol may affect age-associated changes in* C. elegans* such as body movements and intestinal lipofuscin levels. To estimate the healthspan of worms, we recorded travel distance of aged worms (7 days of adulthood) for 20 seconds duration. As can be seen in Figure 6(a), catalpol exposure induced dose-dependent increase in body movement of worms. Surprisingly, 25 *μ*M of catalpol enhanced the total travel distance and average speed of aged worms to over 30% compared to untreated control, suggesting that functional aging of worm is strongly delayed by catalpol (Figure 6(a)). Since, lipofuscin is known as an endogenous marker of cellular damage during aging in many organisms, including* C. elegans* [21], we measured the autofluorescence level of lipofuscin. Our results revealed that fluorescence intensity from intestinal lipofuscin was significantly attenuated in the presence of catalpol by 13.25% at 25 *μ*M (*P* < 0.001, Figure 6(b)).

### 3.5. Mechanistic Studies

**Figure 7:**
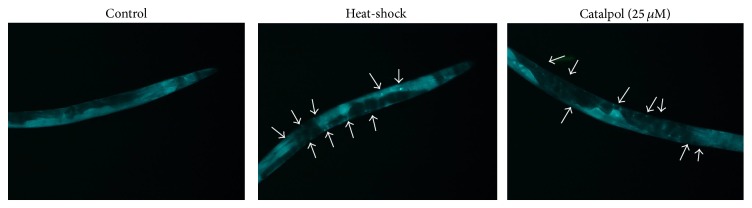
Effects of catalpol on the nuclear localization of DAF-16. The translocation of DAF-16 was visualized under fluorescence microscope using TJ356 strain which carries* daf-16::gfp* transgene. To induce heat-shock, worms were incubated at 36°C for 2 h. Worms were subjected to analyze GFP expression on the 4th day of adulthood.

The underlying mechanism of catalpol-mediated longevity was dissected using loss of function mutant worms relevant to aging [22]. We found that the mean lifespan of sir-2.1 mutants was significantly enhanced by catalpol exposure, indicating that SIR-2.1 is not responsible for catalpol's activity (Table 1). However, catalpol failed to increase the lifespan of mutants including mek-1, daf-2, age-1, daf-16, and skn-1. Thus, we estimated that these genes are involved in catalpol-induced lifespan regulation (Table 1). We double checked the involvement of daf-16 using TJ356 strain which carries* daf-16::gfp* transgene. As can be seen in Figure 7, heat shock triggers DAF-16 nuclear localization and catalpol-fed worms also exhibited similar phenotype, suggesting that catalpol activates the transcriptional activity of DAF-16.

## 4. Discussion

In the current study, we investigated the antiaging activity of catalpol, an active compound of* Rehmannia glutinosa* using* C. elegans* model system. We found that catalpol treatment significantly enhanced the lifespan of wild-type worms under both of normal and stress conditions. In addition, intriguingly, lifespan of worms was prolonged even when the catalpol exposure is started at adulthood. This result suggests that catalpol may give a positive effect on the senescence, a biological aging after maturation, and thus should be an attractive candidate for antiaging drug discovery.

Previous reports have revealed that accumulation of oxidative stress caused ROS is a major factor in aging [23, 24]. Our additional studies suggest that catalpol-induced elevation of antioxidant enzyme activities that resulted in attenuated intracellular ROS might be attributed to extended lifespan. Moreover, oxidative stress promotes accumulation of lipofuscin, a marker of cellular damage during aging, via degeneration of cellular components in many organisms, including* C. elegans* [25]. We demonstrated that the intestinal lipofuscin levels of worms were significantly decreased by catalpol treatment compared to control. Since early studies have suggested that antioxidant treatment prevents lipofuscin [26], our results provide a further evidence that catalpol's antioxidant potential might be contributed to delaying aging.

Herein we also checked whether some alterations in aging-related factors are associated with catalpol-mediated longevity. Reductions of age-related parameters, including reproduction, food intake, and growth, have been known to closely interconnected with longevity in many species [18, 19]. We displayed that catalpol attenuated food intake of worms, while no change was observed in other parameters such as reproduction and growth, indicating that dietary restriction (DR) like effects of catalpol might be possibly linked with lifespan-extension.

Nowadays, the goal of antiaging medicine has been changed from simply extending lifespan to increasing healthspan. Here we showed that catalpol supplementation effectively delayed age-related deterioration of body movement of worms, compared with untreated control, indicating catalpol could enhance healthspan of worms. The* eat-2* (*ad465*) mutant shows dietary restriction phenotype via slowing the rate of pharyngeal pumping [27]. Previous studies suggest that the locomotory ability is highly preserved to older ages of* eat-2* mutant, compared to wild-type worms [28]. Therefore, catalpol may not be responsible for muscle deterioration in the pharynx and resulted in diminished pharyngeal pumping rate but rather modulates age-associated functional decline via DR.

To understand underlying genetic mechanisms by which catalpol extends lifespan, we conducted lifespan assay using several null mutants. Previous studies have indicated that genetic interference by conserved transcriptional factors including DAF-16 and SKN-1 increases lifespan in* C. elegans* [29, 30]. DAF-16, a FOXO-family transcriptional factor, has numerous target genes which confer an enhanced stress resistance and extended lifespan [31]. Our experiments demonstrate that DAF-16 is required for catalpol-mediated lifespan extension. We further investigated whether catalpol accelerates DAF-16 activation using TJ356 strain which carries* daf-16::gfp* transgene. Importantly, we confirmed that catalpol exposure induces nuclear translocation of DAF-16. Indeed, as noted above, catalpol increased the expression of SOD-3, a downstream target of DAF-16. In addition, we found that another transcriptional factor SKN-1 is also involved in catalpol's longevity properties. The* skn-1* gene encodes a worm homolog of the Nrf2 that is critical for oxidative stress resistance and promotes longevity [32]. The SKN-1 is associated with inducing DR response via downstream effector NLP-7, a neuropeptide [33]. Therefore, it is plausible that SKN-1 possibly serves as a target molecule of catalpol for DR response.

Both DAF-16 and SKN-1 have been shown to be inhibited by the insulin/IGF signaling (IIS) pathway in* C. elegans* [34, 35]. To test the possibility whether IIS pathway is responsible for catalpol-mediated activation of these transcriptional factors, we analyzed the lifespan of* daf-2* and* age-1* null mutants. These genes are known to play an important role in IIS pathway by encoding DAF-2/insulin like receptor and AGE-1/phosphoinositide 3-kinase (PI3K), respectively. In this study, we observed no significant extended lifespan of both mutants after catalpol exposure, indicating that catalpol may activate DAF-16 and SKN-1 via inhibition of IIS pathway.

Additional input into DAF-16 regulation is allowed to JNK pathway, a member of the MAPK family. Previous genetic analysis suggests that the JNK pathway also participated in stress resistance and longevity as a positive regulator of DAF-16 in* C. elegans* [36]. Our findings indicate that catalpol failed to increase the lifespan of* mek-1* mutant lacking MEK-1 (MAPKK) in the JNK pathway, suggesting that catalpol may also activate DAF-16 via regulation of JNK signaling, independent of IIS pathway.

SIR-2.1, a family of NAD^+^-dependent histone deacetylases, is another evolutionary conserved regulator of longevity. Previous studies have revealed that overexpression of SIR-2.1 can increase the lifespan of* C. elegans* through either downregulation of IIS pathway or direct activation of DAF-16 in a parallel with IIS signaling [37, 38]. In this study, the possible involvement of SIR-2.1 was also investigated using* sir-2.1* null mutants. Our observation shows that catalpol significantly prolonged the lifespan of* sir-2.1* silenced worms, suggesting that catalpol's longevity activities are independent of regulation of SIR-2.1.

## Figures and Tables

**Table 1:** Effects of catalpol on the lifespan of mutant *C. elegans*.

Genotype	Mean lifespan^a^		Maximum lifespan		Change in mean lifespan^c^ (%)	Log-rank test^d^
	Untreated	Treated^b^	Untreated	Treated^b^		
Wild-type	12.3 ± 0.3	15.8 ± 0.4	18	27	28.5	P < 0.001^***^
*skn-1 (zu67) *	11.8 ± 0.3	11.7 ± 0.3	20	19	−0.8	*P = 0.595 *
*daf-16 (mgDf50) *	11.7 ± 0.4	11.8 ± 0.4	19	19	0.9	*P = 0.611 *
*daf-2 (e1368) *	16.1 ± 0.8	16.1 ± 0.7	27	26	0.0	*P = 0.951 *
*age-1 (hx546) *	15.2 ± 0.5	15.7 ± 0.6	28	29	3.4	*P = 0.356 *
*sir-2.1 (ok434) *	11.4 ± 0.6	12.6 ± 0.7	19	23	10.3	P = 0.010^*^
*mek-1 (ks54) *	10.2 ± 0.3	9.9 ± 0.3	18	17	−1.1	*P = 0.803 *

^a^Mean lifespan presented as mean ± S.E.M.

^b^Catalpol-treated concentration was 25 *μ*M.

^c^Change in mean lifespan compared with untreated group of each strain (%).

^d^Statistical significance of the difference between survival curves was determined by log-rank test using the Kaplan-Meier survival analysis. Differences compared to the control were considered significant at ^*^ *P* < 0.05 and ^***^ *P* < 0.001.
